# The Mechanisms of Yu Ping Feng San in Tracking the Cisplatin-Resistance by Regulating ATP-Binding Cassette Transporter and Glutathione S-Transferase in Lung Cancer Cells

**DOI:** 10.3389/fphar.2021.678126

**Published:** 2021-05-28

**Authors:** Yingqing Du, Yuzhong Zheng, Ciel Xiaomei Yu, Lishan Zhong, Yafang Li, Baomeng Wu, Weihui Hu, Elsa Wanyi Zhu, Venus Wei Xie, Qitian Xu, Xingri Zhan, Yamiao Huang, Liyi Zeng, Zhenxia Zhang, Xi Liu, Jiachuan Yin, Guangcai Zha, Kelvin Chan, Karl Wah Keung Tsim

**Affiliations:** ^1^ Guangdong Key Laboratory for Functional Substances in Medicinal Edible Resources and Healthcare Products, School of Life Sciences and Food Engineering, Hanshan Normal University, Chaozhou, China; ^2^ Division of Life Science, Center for Chinese Medicine, The Hong Kong University of Science and Technology, Kowloon, China; ^3^ School of Pharmacy and Biomolecular Science, Liverpool John Moores University, Liverpool, United Kingdom; ^4^ United Kingdom and NICM Health Research Institute, Western Sydney University, Sydney, NSW, Australia

**Keywords:** yu ping feng san, cisplatin, GSTs, efflux transporters, anti-multidrug resistance, prim-o-glucosylcimifugin

## Abstract

Cisplatin is one of the first line anti-cancer drugs prescribed for treatment of solid tumors; however, the chemotherapeutic drug resistance is still a major obstacle of cisplatin in treating cancers. Yu Ping Feng San (YPFS), a well-known ancient Chinese herbal combination formula consisting of Astragali Radix, Atractylodis Macrocephalae Rhizoma and Saposhnikoviae Radix, is prescribed as a herbal decoction to treat immune disorders in clinic. To understand the fast-onset action of YPFS as an anti-cancer drug to fight against the drug resistance of cisplatin, we provided detailed analyses of intracellular cisplatin accumulation, cell viability, and expressions and activities of ATP-binding cassette transporters and glutathione S-transferases (GSTs) in YPFS-treated lung cancer cell lines. In cultured A549 or its cisplatin-resistance A549/DDP cells, application of YPFS increased accumulation of intracellular cisplatin, resulting in lower cell viability. In parallel, the activities and expressions of ATP-binding cassette transporters and GSTs were down-regulated in the presence of YPFS. The expression of p65 subunit of NF-κB complex was reduced by treating the cultures with YPFS, leading to a high ratio of Bax/Bcl-2, i.e. increasing the rate of cell death. Prim-O-glucosylcimifugin, one of the abundant ingredients in YPFS, modulated the activity of GSTs, and then elevated cisplatin accumulation, resulting in increased cell apoptosis. The present result supports the notion of YPFS in reversing drug resistance of cisplatin in lung cancer cells by elevating of intracellular cisplatin, and the underlying mechanism may be down regulating the activities and expressions of ATP-binding cassette transporters and GSTs.

## Introduction

Lung cancer is the most diagnosed cancer with overwhelming mortality among all types of cancers, which can be classified into non-small-cell lung cancer (NSCLC) and small-cell lung cancer (SCLC). NSCLC accounts for over 85% of total lung cancer incidents (Siegel et al., 2014). In cancer chemotherapy, most of the anti-cancer drugs exhibit resistance by cancer cells, and which results in failure of chemotherapy and/or tumor recurrence. Multidrug resistance (MDR) is a major impediment to chemotherapeutic efficacy in cancer patients. At the beginning of chemotherapeutic treatment, intrinsic drug resistance is causing unresponsiveness of cancer cell to chemotherapy. The acquired resistance arises in recurrence of tumor, and which thereafter displays resistance to a broad range of structural and functional diverse chemicals. The transformation of tumor with MDR could be mediated by various parameters, e.g. regulating drug efflux/influx, changing drug detoxification system, expressing gene/protein in apoptosis and drug distribution (Larsen et al., 2000; Baird and Kaye, 2003).

Cisplatin, a platinum-based anti-cancer drug with known action mechanism and at low cost, has been widely used in chemotherapy for a variety of tumors. The cytotoxic action of cisplatin is to interact with DNA forming DNA adducts, and ultimately triggers cell apoptosis (Siddik, 2003). However, the clinical usage of cisplatin is limited by its severe dose-limiting side effect and MDR (Chen et al., 2019). Combining cisplatin with other anti-cancer drugs during chemotherapy therefore is the cornerstone in treating various cancers, e.g. lung cancer. Tackling the reason of cisplatin-resistance in cancer is of great significance.

Glutathione S-transferases (GSTs), a large family of isozymes, are multi-functional enzymes having crucial roles in cellular detoxification and oxidative stress tolerance. The most important members of GSTs are GSTPi1, GSTM1, GSTM2, and GSA1 (Ekhart et al., 2009). GSTs catalyze the nucleophilic addition of glutathione (GSH) to non-polar exogenous, e.g. cisplatin, yielding water-soluble conjugates. The mono-hydrated form of cisplatin is a highly reactive species, and which is a rate-limiting step to interact with endogenous nucleophiles, e.g. GSH, forming a variety of non-toxic cisplatin conjugates (Siddik, 2003). The GSH-conjugated cisplatin can be transported by multidrug resistance associated protein 2 (MRP2), as a result of low bioavailability, leading to cisplatin-resistance (Siddik, 2003; Wen et al., 2014). The upregulation and hyperactivation of these GSTs could enhance the catalytic detoxification of anti-cancer drugs and modulate apoptotic signaling, and indeed the level of GSTs is being considered as one of the major causes of MDR in cancer cells (Singh, 2015). Glutathione metabolic signaling leads to a decrease of cisplatin being accumulated and developed resistance. Therefore, the suppression of GST activity is expected to enhance the efficacy of cytotoxic agents (Zou et al., 2019).

P-gp (P-glycoprotein), BCRP (breast cancer resistance protein) and MRPs (multidrug resistance associated proteins, including MRP1, MRP2, and MRP3) are known members of ATP-binding cassette (ABC) efflux transporters. Most of the anti-cancer drugs are substrates of these transporters that efflux transport the drugs resulting in an ATP-dependent decrease of intracellular drug accumulation. Thus, MDR is usually considered as a severe consequence of overexpression and hyperactive of ATP-binding cassette efflux transporters (Leslie et al., 2005). An inhibition of efflux transporter is expected to be one of the effective ways to re-sensitize MDR cells during chemotherapy.

Yu Ping Feng San (YPFS) is an ancient Chinese herbal decoction described in “Dan Xi Xin Fa” by Zhu Danxi (A.D. 1279–1368) (Wilcox, 2004), one of the four great masters in using herbal treatments on damp-heat and yin-insufficiency conditions in traditional Chinese medicine (TCM) during the Jin-Yuan Dynastic period. YPFS composes of Astragali Radix [AR, Huangqi, the root of *Astragalus membranaceus* (Fisch.) Bunge or *Astragalus membranaceus* (Fisch.) Bunge var. *mongholicus* (Bunge) P. K. Hsiao], Atractylodis Macrocephalae Rhizoma (AMR, Baizhu, the rhizomes of *Atractylodes macrocephala* Koidz.) and Saposhnikoviae Radix [SR, Fangfeng, the roots of *Saposhnikovia divaricata* (Turcz.) Schischk.] in a weight ratio of 1:2:1 (Du et al., 2013). Our previous study demonstrated that YPFS modulated inflammatory cytokines and enzymes to exert immune functions (Du et al., 2013; Du et al., 2014; Du et al., 2015), and regulated p62/TRAF6 signaling and WT1-mediated stabilization on mTOR/AKT axis in both cell and animal models (Du, 2014; Lou et al., 2016; Lou et al., 2018). The underlying mechanism of anti-drug resistance efficacy of YPFS in a fast-onset manner however has not yet been elucidated. Here, we demonstrated the fast action of YPFS in reversing the cisplatin-resistance in cultured adeno-carcinomic human alveolar basal epithelial cells (A549) and its cisplatin-resistance cells (A549-DDP). In parallel, the activities of GSTs and ATP-binding cassette efflux transporters were measured, and the apoptotic related targets, i.e. p65, Bax and Bcl-2, were investigated here. The active role of prim-O-glucosylcimifugin, an identified active ingredient in YPFS, in fighting against cisplatin resistance was further revealed.

## Materials and Methods

### Herbal Preparation

The roots of *A. membranaceus* var. *mongholicus* (AR), the rhizomes of *A. macrocephala* (AMR) and the roots of *S. divaricata* (SR), purchased from medicinal herbal market in Puning City, Guangdong Province, were authenticated morphologically by one of the authors, Dr. Du. The herbs, standardized and met the quality control standard of Chinese Pharmacopoeia, were utilized for YPFS preparation. The corresponding voucher specimens, voucher AR18-01, voucher AMR18-01, and voucher SR18-01, in the form of whole plants, were deposited at Room 710 of Like Building at Hanshan Normal University. No specific permission was required for the location or activity during the collection of raw material. The location was not privately owned or protected. The herbal extracts of YPFS were prepared as the method established by our previously study (Du et al., 2013). Briefly, the herbs of AR, AMR and SR in a weight ratio of 1:2:1 were boiled together in eight volumes of water (v/w) by moderate heating for 2 h, and then the herbal residues were re-boiled in six volumes of water for 1 h. All decoctions were combined, filtered and lyophilized, and then stored at 4°C. Here, a standardized YPFS was calibrated by a method described before (Du et al., 2013). A gradient elution of 5–95% acetonitrile at 0–80 min and 210 nm detection wavelength were set as an optimized parameter. The herbal extracts were dissolved in water at 50 mg/ml for 30 min sonication, and which were filtered through 0.22 μm filtration membrane before high performance liquid chromatography (HPLC) injection. SB-C18 analytical HPLC column was used for separation of YPFS in an Agilent rapid resolution HPLC 1200 system. The amount of chemicals in YPFS was then determined, meeting the minimum requirements of a standardized YPFS according to previous study (Du et al., 2013).

### Chemicals

The chemical markers (purity ≥98%) were purchased from Sichuan Victory Biological Technology Co., Ltd. Materials for tissue culture were purchased from Invitrogen (Carlsbad, CA). The antibodies were obtained from Abcam (Cambridge, United Kingdom), Absin (Absin Bioscience Co., Ltd.) and Invitrogen. BD GentestTM ATPase assay kit was purchased from BD Bioscience (San Jose, CA). GST activity assay kit, BCA protein assay kit, A549 cell lines were purchased from Dingguo Changsheng Biotechnology Ltd. (Guangzhou, China). A549-DDP (cisplatin resistance) cell was purchased from Fudan IBS cell bank (FDCC). TRIzol^™^ Reagent, High-Capacity cDNA Reverse Transcription Kits, PowerUp^™^ SYBR^™^ Green Master Mix, Pierce^®^ Fast Western Blot Kit, and PVDF Transfer Membrane were purchased from ThermoFisher Scientific (Guangzhou, China). Materials that not specified here were purchased from Sigma-Aldrich. (St. Louis, MO), ThermoFisher Scientific, Merck (Whitehouse Station, NJ), Dingguo and Xinjing Biotechnology.

### Cell Culture

A549 and A549-DDP cells were cultured in RPMI-1600 supplemented with 10% fetal bovine serum and 100 U/ml of penicillin and 100 μg/ml of streptomycin, and which were maintained at 37°C in a humidified atmosphere of 5% CO_2_. A549-DDP cells were maintained in the presence of 2.5 µM cisplatin, which were withdrawn for two generations before drug treatment. Ninety percent confluency of cells grown in the logarithmic phase were washed with PBS twice, and then cells were harvested by using 0.25% trypsin-EDTA about 40 s for cell dissociation before the sub-culture. After seeding, the cells were cultured overnight prior to drug treatment.

### Cellular Accumulation of Cisplatin

Cells were plated in 100-mm culture plates and cultured to 90% confluent, then the medium was replaced with fresh medium containing 80 µM cisplatin in the absence or presence of YPFS at concentrations of 0–3 mg/ml, or MK571 at concentrations of 0–12 μM, or prim-O-glucosylcimifugin at 0–10 μM, for 3 h before the measurement of cisplatin being accumulated inside the cells. After 3 h of drug treatment, the cell monolayers were washed three times with PBS. The cells were then harvested by a scraper and were removed from plates by PBS. The cell suspension was centrifuged at 1,000× *g* for 3 min at 25°C. Then, the cell pellets were lyophilized, and the total amount of the protein was measured. The lyophilized cell pellets were mineralized in 500 µl 70% HNO_3_ spiking with 40 μg/L cadmium at 80°C for 2 h, and then diluted with 2.5 ml H_2_O. Thermo Scientific MaxQ 4,000 benchtop refrigerated shaker was used for sample digestion. Platinum determination was performed with inductively coupled plasma-optical emission spectroscopy (ICP-OES). The amount of platinum was corrected in respect to cadmium signal and total protein.

### Cell Viability Assay

Five thousand cells per well were seeded in 96-well plate overnight, and then treated with various concentration of cisplatin, or 2.5 µM cisplatin in present or absent of YPFS at concentrations of 0–3 mg/ml, or prim-O-glucosylcimifugin at 0–10 µM for 24 h in cultured A549 or A549-DDP cells. The group without drug treatment was taken as a control. Subsequently, 10 µl MTT (5 mg/ml) was added into the culture for another 1 h. Then, the medium was removed, and DMSO solvent was added to extract purple formazan. The absorbance at 570 nm was measured. The percentage of cell apoptosis was calculated by the means of optical density (OD) in the indicated group in six repeats. Cell apoptosis rate (%) = 1—(OD_treatment group_/OD_control group_) × 100%.

### Glutathione S-transferase Activity

Forty thousand cells per well were seeded in 12-well plate overnight, and then were treated with YPFS at concentrations of 0–3 mg/ml, or prim-O-glucosylcimifugin at concentrations of 0–10 µM. After 48 h of culture, the cells were then washed and scraped with pre-chilled PBS at a ratio of cell number (10^6^): PBS (µl) = 1 : 300–500. The collected cells were then lyzed by ultrasonic breaker in ice water bath for 2 s sonication and 3 s interval within 5 min of total time under 200 W, and which were centrifuged at 10,000 × *g* for 10 min. The supernatant was preserved on ice. In accord to Elabscience GST activity assay kit, the reagent three buffer and cuvette were pre-heated at 37°C for 10 min and spectrophotometer was set to zero with double distilled water. One hundred μl cell lysate was added to mix buffer, according to instruction. Absorbance at 340 nm at 20 s (A_1_) and 320 s (A_2_) were obtained, respectively. ΔA was obtained from A_2_ subtracted A_1_. One unit of GST activity was defined as the amount of GST in 1 mg of tissue protein that catalyzes the combination of 1 µM of CDNB and GSH at 37°C per min.

$$GSTactivity\;\left(U/mL\right)=\Delta A/\left(\varepsilon \times d\right)\times 10^{6}÷t\times \left(V_{1}/V_{2}\right)\times f÷Cpr,$$ (1)

ε: molar extinction coefficient of the product, 9.6 × 10^3^ L/mol/cm,
d: optical path of the cuvette, 1 cm,
10^6^: 1 mol = 10^6^ μmol,
V_1_: the total volume of the reaction system, 1.1 ml,
V_2_: the volume of sample added into the reaction system, 0.1 ml,
T: reaction time, 5 min,
C_pr_: concentration of protein in sample, mg/mL.

For investigating direct effect of YPFS on GST activity, the lysates from non-treated cells were obtained and incubated with YPFS for 5 min at 37°C, and then GST activity was assayed as described above in parallel.

### ATPase Activity

The plasma membrane was diluted to 1 mg/ml by using the assay buffer. Then, the membrane was pre-incubated with or without YPFS about 5 min. Sodium orthovanadate (NaOV) inhibits ATPase by trapping Mg^2+^ADP in the nucleotide-binding sites. Thus, each identical reaction mixture containing NaOV (0.25 mM for P-gp, 1 mM for BCRP, MRP1 and MRP2, 3 mM for MRP3) was assayed for further 10 min in parallel. Probe substrates, i.e. 20 µM verapamil for P-gp 10 µM sulfasalazine for BCRP, 10 mM (NEM:GSH) complex for MRP1, 1 mM probenecid for MRP2 and 50 µM benzbromarone for MRP3, accompany with 4 mM Mg^2+^ATP solution, were added to activate ATPase activity, separately. The corresponding incubation time for activation of ATPase activity assay was 10 min for BCRP, 20 min for P-gp, 40 min for MRP2, 60 min for MRP1 and MRP3, separately. Here, ATPase activity measured with NaOV represented non-ATPase activity, and which was subtracted from the total activity measured in the sample to yield ATPase activity. The reaction was stopped by addition of 10% SDS, and liberation of inorganic phosphate was detected by colorimetric reaction with ascorbic acid in ammonium molybdate solution (Drueckes et al., 1995).

### Western Blot Analysis

Cells were washed with pre-chilled PBS, three times, lyzed with RIPA and vortexed for 10 min, and then centrifuged at 10,000 × *g* for 10 min at 4°C. Total protein was measured by BCA protein assay kit and diluted to 1 mg/ml. Thirty μg protein was subjected to sodium dodecyl sulfate-polyacrylamide gel electrophoresis (SDS-PAGE), and then were transferred to a PVDF membrane from polyacrylamide gels at 90 mA for 20 h in 1 × transfer buffer containing 25 mM Tris, 192 mM glycine, 15% ethanol and 0.1% SDS. The membrane was blocked with 5% non-fat milk in 20 mM Tris base, 137 mM NaCl, 0.1% Tween-20, pH 7.6 (TBS-T) for 1 h at 4°C followed by incubation with primary antibody overnight with gentle agitation. The dilutions for all primary antibodies are at 1:1,000, and 1:5,000 for GAPDH. After intensive washing with TBS-T, horseradish peroxidase (HRP)-conjugated goat anti-mouse, or anti-rabbit secondary antibody, with 1:5,000 dilution was added and incubated for 2 h at 4°C. The protein signal was developed by ECL method and measured by using a FluorChem HD2 system (Alpha Innotech).

### Ribonucleic Acid (RNA) Extraction and Quantitative Real-Time Polymerase Chain Reaction (PCR) 

Total RNA was isolated from A549/A549-DDP cells using TRIzol (Invitrogen). Synthesis of cDNA was performed using 2 μg of total RNA using high capacity cDNA Reverse Transcription Kits. Intron-spanning primer was designed to amplify by real-time quantitative PCR. The sequences were listed as follow: Bax: 5′- TGA CGG CAA CTT CAA CTG-3′, 3′-CTT CCA GAT GGT GAG TGA G-5′, Bcl-2: 5′-TGT GGA TGA CTG AGT ACC T-3′, 3′-CAG AGA CAG CCA GGA GAA- 5′, GSTPi1: 5′-TAC CAG TCC AAT ACC ATC CT-3′, 3′- GCC TTC ACA TAG TCA TCC TT-5′, GSTM1: 5′-GAC GCT CCT GAT TAT GAC A-3′, 3′-CAC ACG AAT CTT CTC CTC TT-5′, GAPDH: 5′-TAT GAC AAC AGC CTC AAG AT-3′, 3′-AGT CCT TCC ACG ATA CCA-5’. Quantitative real-time PCR analysis was performed with 2 × PowerUp^™^ SYBR^™^ Green Master Mix, using Roche LightCycler^®^ 96 Detection System. The quantitative real-time PCR parameter was as follows: 2 min at 50°C for UDG activation and 2 min at 95°C for Dual-Lock^™^ DNA Taq polymerase enzyme to initiate hot-start mechanism, then 40 cycles of denaturation at 95°C for 15 s, annealing at 55°C for 15 s, and extension at 72°C for 1 min. The gene expression level was normalized to that of GAPDH in each sample and calculated as the threshold cycle (CT) value in each sample, divided by the CT value in each reference.

### Statistical Analysis

Data are presented as Mean ± SEM (standard error of mean). Statistical analysis was performed with SPSS 22.0 statistical software (SPSS, Chicago, IL). The statistical difference among groups was analyzed by using one way-ANOVA. *p* value less than 0.05 was considered as a statistically significant difference.

## Results

### Yu Ping Feng San Increases Intracellular Cisplatin

A standardized YPFS was prepared, as described in our previous study (Du et al., 2013). A typical HPLC profile of YPFS containing various known chemical markers, as well as those of single herbs AR, AMR and SR, were shown (Supplementary figure S1). Here, 1 g of prepared YPFS contained 0.533 mg/g calycosin-7-O-β-D-glucoside, 0.251 mg/g calycosin, 0.701 mg/g ononin, 0.378 mg/g formononetin, 0.145 mg/g astragaloside IV, 0.120 mg/g astragaloside III, 0.086 mg/g astragaloside II, 0.042 mg/g atractylenolide I, 0.073 mg/g atractylenolide II, 0.382 mg/g atractylenolide III, 0.882 mg/g prim-O-glucosylcimifugin, 0.588 mg/g 5-O-methylvisammioside, 0.516 mg/g cimifugin and 0.307 mg/g sec-O-glucosylhamaudol. The amounts of chosen marker chemicals in the prepared YPFS met the chemical parameters established before (Du et al., 2013).

**FIGURE 1:**
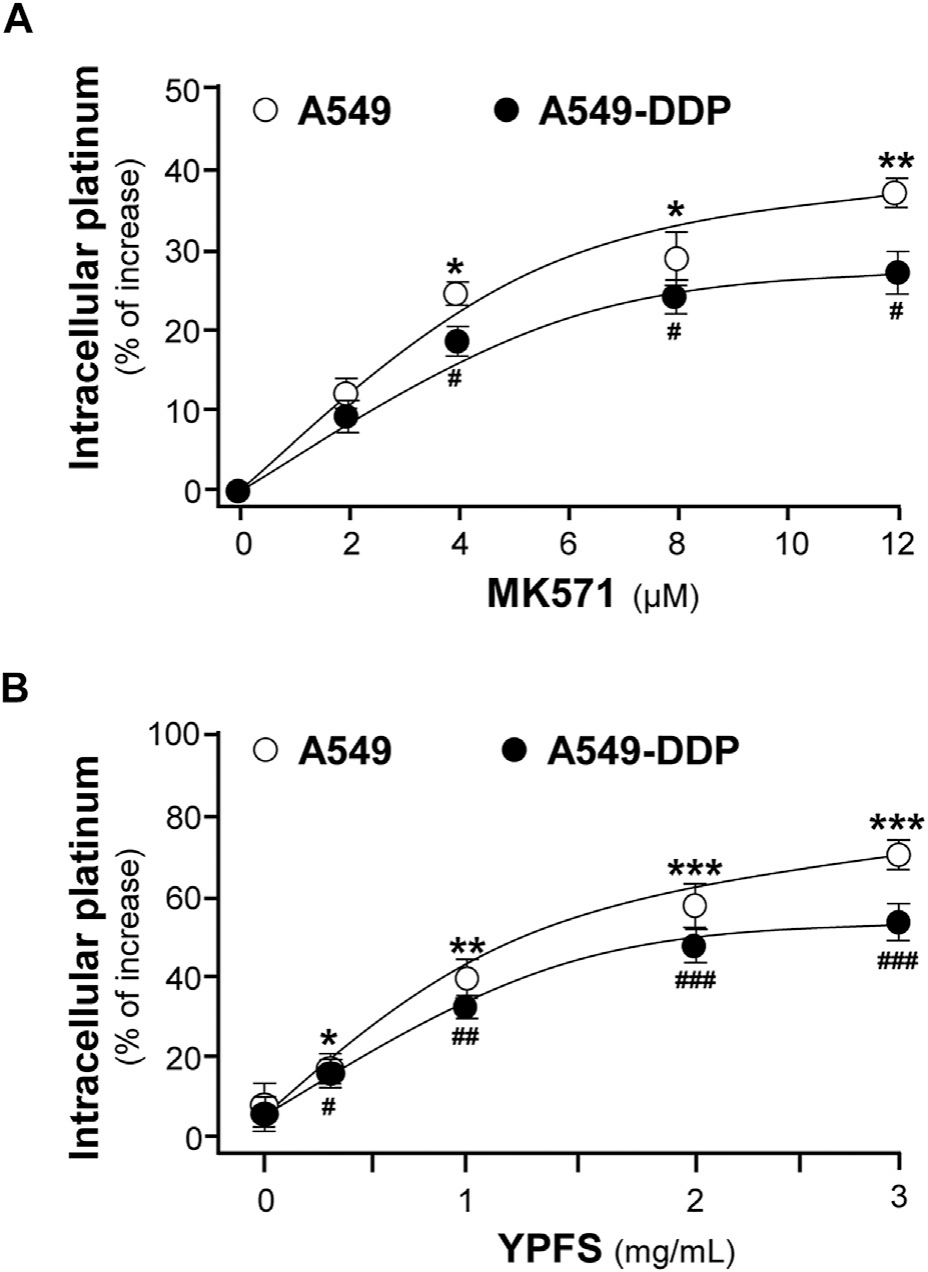
YPFS increases intracellular platinum in cultured A549 and A549-DDP cells. Cultured A549 and A549-DDP cells (500,000) were plated in a 100-mm culture plate and cultured to 90% confluent. Then, cells were challenged with 2.5 µM cisplatin (non-toxic dose) and MK 571 **(A)** or YPFS extract **(B)** for 3 h. After washing, the collected cells were digested with 500 μl 70% HNO_3_ spiked with 40 μg/ml cadmium for 2 h and adjusted with water to 3 ml and subjected to ICP-OES analysis. Values are normalized with total protein and cadmium signal, expressed as % of increase as compared with control group (no drug), in mean ± SEM, *n* = 3. * or ^#^, *p* < 0.05, ** or ^##^, *p* < 0.01, *** or ^###^, *p* < 0.001.

In contrast to A549 cells, A549/DDP cells showed drug resistance to cisplatin. Our previous study has shown that YPFS could increase intracellular accumulation of the active ingredients, i.e. calycosin and formononetin, within 3 h in the cultures: these compounds were the substrates of efflux transporters, as that of cisplatin (Du, 2014). Thus, we hypothesized that the synergy of YPFS with cisplatin in inducing toxicity of cancer cells could be related to GST and efflux drug transporter. To test this hypothesis, an ICP-OES method was developed for quantification of intracellular platinum, i.e. the key element in cisplatin. MK571, an inhibitor for efflux transporter, served as a positive control. MK571 at different concentration was applied onto the cultures to test the efficiency in accumulating intracellular platinum: the maximal effect was up to ∼35 or ∼25% in cultured A549 or A549-DDP cells, respectively, as compared with control group (Figure 1A). The applied YPFS increased intracellular platinum in a dose-dependent manner (Figure 1B), similar to that of MK571. The robust effect of YPFS in increasing intracellular platinum, within 3 h, was up to 70 and 50% in cultured A549 and A549-DDP cells, respectively (Figure 1B). The results indicated that YPFS could increase accumulation of intracellular cisplatin in cultured A549 and A549-DDP cells in a fast-onset manner.

**FIGURE 2:**
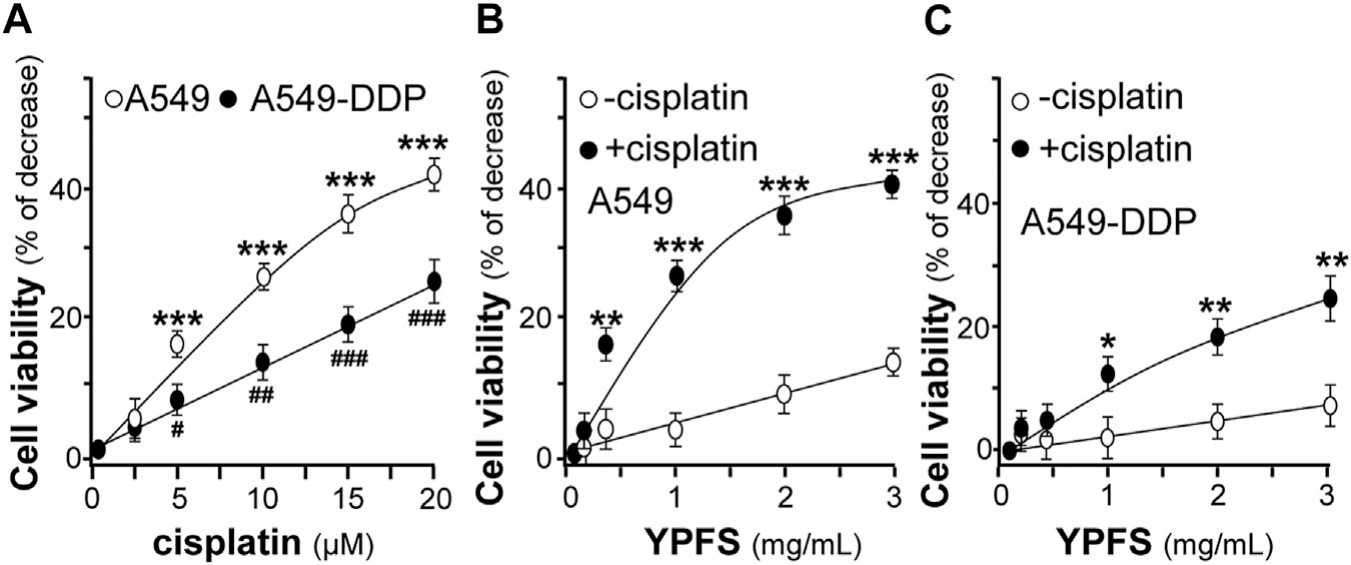
YPFS potentiates cytotoxic activity of cisplatin in cultured A549 and A549-DDP cells. **(A)**: The cytotoxic activity of cisplatin against cultured A549/A549-DDP cells **(B):** Synergy of YPFS and cisplatin in cytotoxic activity in cultured A549 cells. **(C):** Synergy of YPFS and cisplatin in cytotoxic activity in cultured A549-DDP cells. A549/A549-DDP cells in a logarithmic phase were seeded (5,000/well) in 96-well plates and allowed to adhere overnight. Cells were then treated with drugs for 48 h. Cisplatin at 2.5 µM (non-toxic dose) was co-applied with YPFS to test the synergistic effect in cell viability. MTT assay was applied to determine the cytotoxic activity. Values are expressed as % of increase, as compared to control group (no drug), in mean ± SEM, *n* = 3. * or ^#^, *p* < 0.05, ** or ^##^, *p* < 0.01, *** or ^###^, *p* < 0.001.

The potentiating effect of YPFS in cytotoxicity of cisplatin in cultured A549 and A549-DDP cells was determined. Cisplatin at 20 μM increased the rate of cytotoxicity up to ∼43% for A549 cells and ∼27% for A549-DDP cells. Meanwhile, cisplatin at 2.5 μM did not show any cytotoxicity (Figure 2A). Thus, cisplatin at 2.5 μM without toxicity was applied onto cultured A549, or A549-DDP, cells in present or absent of YPFS for 24 h, as to determine the synergy in cell viability. YPFS by itself at high concentration showed toxicity in the cultures (Figures 2B,C). The co-applied YPFS showed synergy in potentiating the toxicity of cisplatin at 2.5 μM (a non-toxic dose) in a dose-dependent manner. The cytotoxicity of YPFS accompany with cisplatin was increased up to four folds in cultured A549 cells (Figure 2B). Similar potentiating effect of YPFS with cisplatin was found in cultured A549-DDP cells, over two folds as that of YPFS only (Figure 2C).

### Yu Ping Feng San Suppresses Glutathione S-transferase and ATPase

**FIGURE 3:**
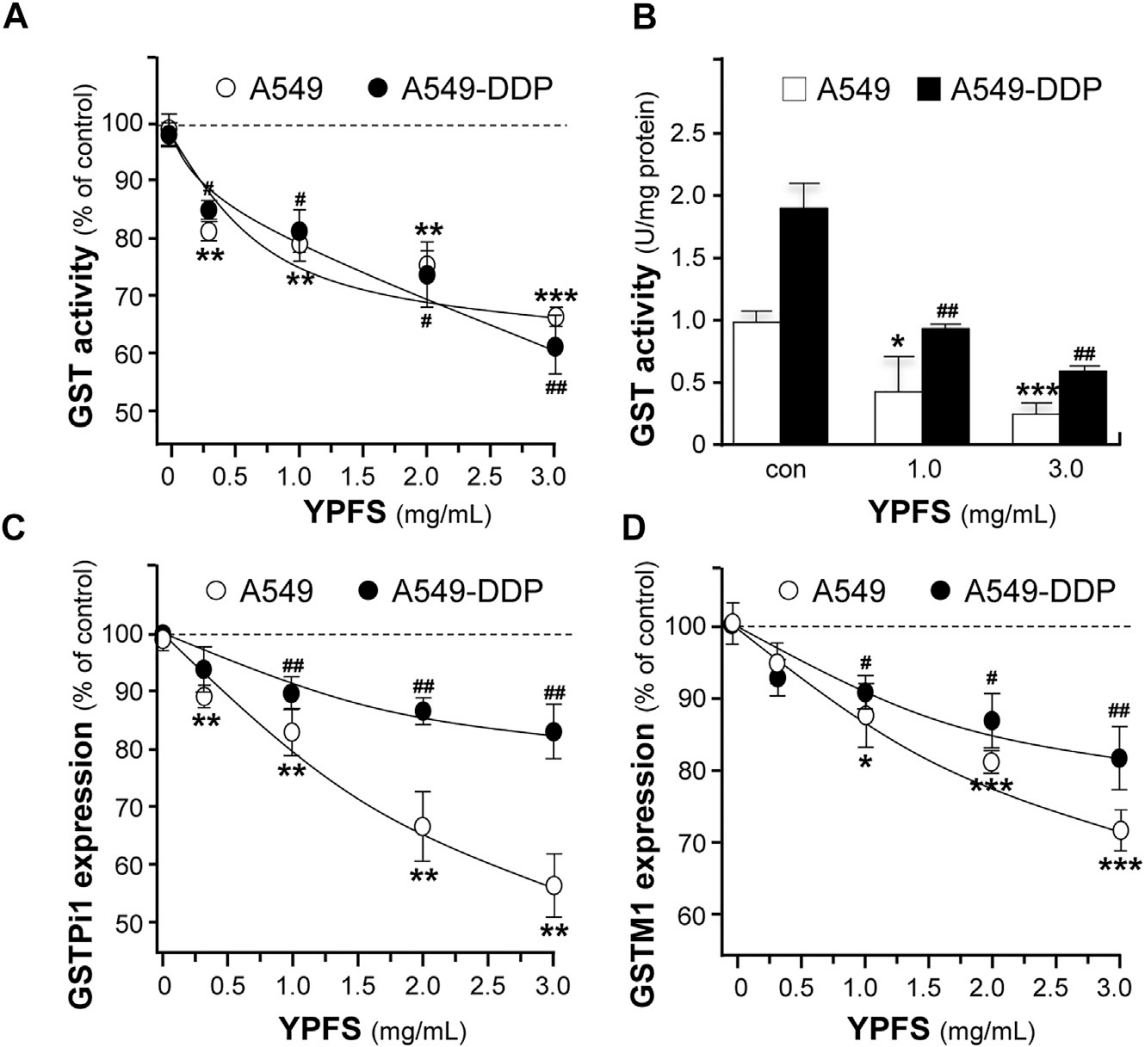
YPFS suppresses GST activity and expression in cultured A549 and A549-DDP cells. **(A)**: Cultured A549 and A549-DDP cells (40,000/well in 12-well plate) were treated with YPFS for 48 h, and then the activity of GST was determined. **(B)**: YPFS was applied to A549/A549-DDP cell lysate, and then GST activity was measured. Values are expressed as unit of GST activity. **(C and D)**: Cultured A549 and A549-DDP cells (120,000/well in 6-well plate) were treated with YPFS for 24 h, and then mRNA expression levels of GSTPi1 **(C)** and GSTM1 **(D)** were determined using quantitative real-time PCR. GAPDH was used as an internal control for normalization. Values are expressed as % of control (no drug), in mean ± SEM, *n* = 3. * or ^#^, *p* < 0.05, ** or ^##^, *p* < 0.01, *** or ^###^, *p* < 0.001.

The level of GST activity was evaluated in cultured A549 and A549-DDP cells, in the presence of YPFS for 48 h. The presence of YPFS alone in the cultures decreased the activity of GST dose-dependently: the effects were ∼30% in A549 cells and ∼40% in A549-DDP cells (Figure 3A). The decreased GST activity might be due to two reasons, i.e. inhibition of GST activity or reduction of GST protein expression. YPFS was directly applied onto the cell lysates, and then GST activity was inhibited (Figure 3B). In addition, the expressions of transcripts encoding different members of GSTs, e.g. GSTPi1 and GSTM1, were measured by real-time PCR. The presence of YPFS effectively decreased GSTPi1 expression, up to ∼55 and ∼20% of decrease in cultured A549 and A549-DDP cells, respectively (Figure 3C). In parallel, YPFS decreased the expressions of GSTM1 at ∼30% (A549) and at ∼20% (A549-DDP) in dose-dependent manners (Figure 3D). Thus, YPFS could reduce both expression and activity of GSTs, and which consequently reduced the soluble form of cisplatin-conjugate, i.e. inhibiting extrusion of cisplatin being pumped out of the cell.

**FIGURE 4:**
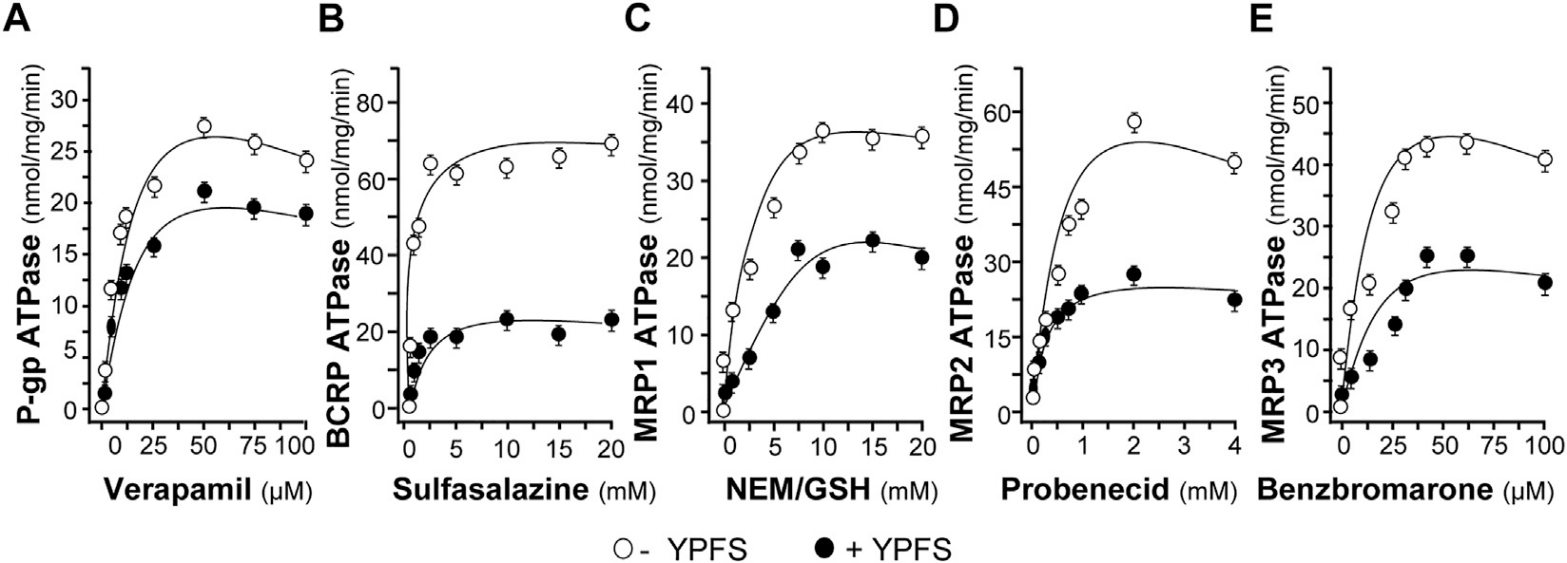
YPFS suppresses ATPase activity. YPFS suppressed verapamil-stimulated P-gp ATPase **(A)**, sulfasalazine-stimulated BCRP ATPase **(B)**, NEM/GSH-stimulated MRP1 ATPase **(C)**, probenecid-stimulated MRP2 ATPase **(D)** and benzbromarone-stimulated MRP3 ATPase **(E)**. The ATPase substrate was added to plasma membranes, with or without 3 mg/ml YPFS, for 5 min. Then, Mg^2+^ATP (4 mM) was treated for further incubation, i.e. 20 min for P-gp, 10 min for BCRP, 60 min for MRP1, 40 min for MRP2 and 60 min for MRP3. An identical reaction mixture containing 0.250 mM NaOV was assayed in parallel. Thus, ATPase activity measured in the presence of NaOV represented non-ATPase activity, and which would be subtracted from the total activity measured in the various samples to yield ATPase activity. The reaction was stopped by the addition of 10% SDS and liberation of inorganic phosphate was detected by colorimetric reaction with ascorbic acid in ammonium molybdate solution. The amount of inorganic phosphate released by 1 mg ATPase within 1 min represents enzyme activity. Mean ± SEM, *n* = 3.

To substantiate the role of YPFS in protein-drug interaction, we performed the assay of ATPase activity. Verapamil, a substrate for P-gp ATPase, was used to stimulate the enzymatic activity, with or without 3 mg/ml YPFS, for 5 min. The activity of verapamil-induced P-gp ATPase was suppressed by YPFS (Figure 4A). In parallel, the suppressive effects of YPFS in ATPase substrates, e.g., sulfasalazine for BCRP ATPase, NEM/MSH for MRP1 ATPase, probenecid for MRP2 ATPase and benzbromarone for MRP3 ATPase, were revealed (Figures 4B–E). The results suggested that YPFS could reduce ATPase activity of the efflux transporters. Cisplatin is known to be efflux transported by MRP2 *via* activating the ATPase activity. Here, YPFS application was shown to decrease the MRP2 ATPase activity, which therefore could affect the cisplatin transport, as mediated by MRP2 ATPase. The result was in accord to the fast-onset of YPFS in increasing intracellular accumulation of cisplatin.

### Yu Ping Feng San Suppresses Expressions of P-glycoprotein, Breast Cancer Resistance Protein and p65

**FIGURE 5:**
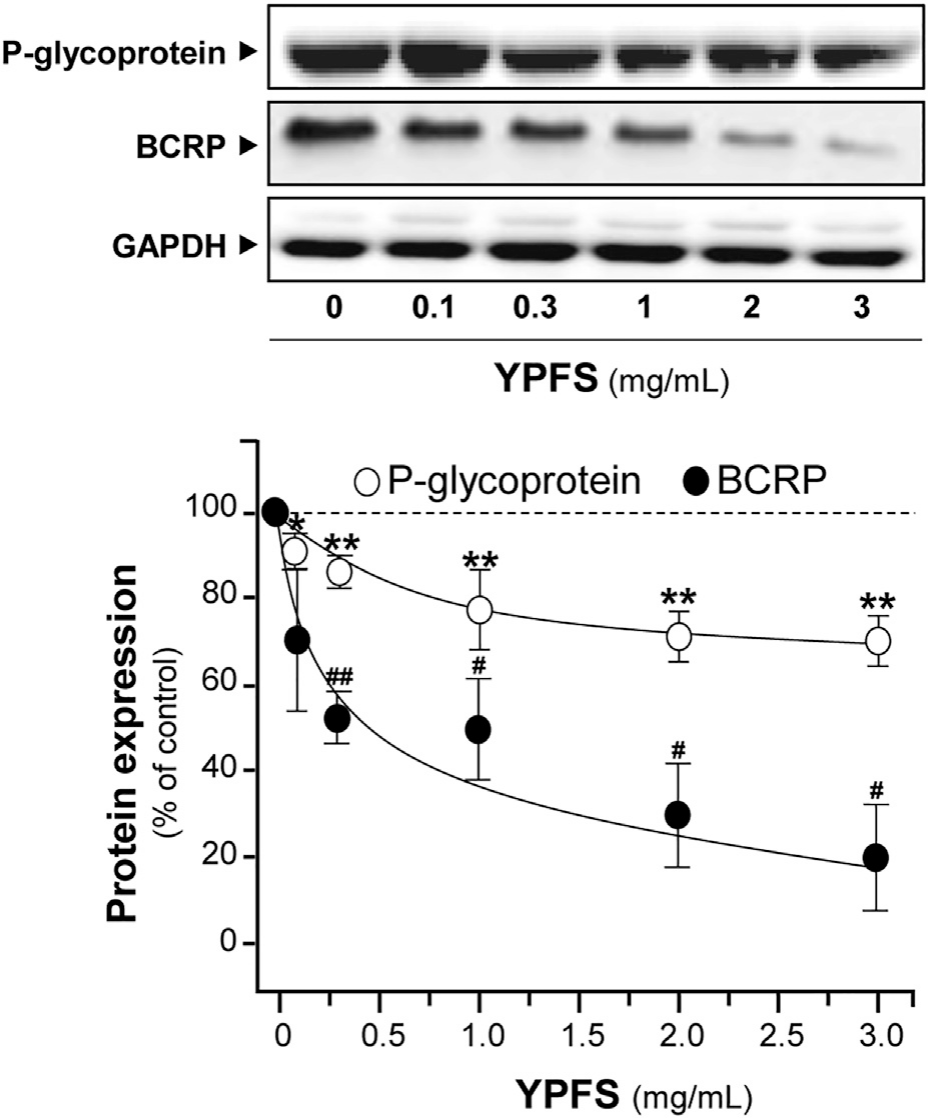
YPFS suppresses protein expressions of P-gp and BCRP in cultured A549-DDP cells. Cultured A549-DDP cells (120,000/well in 100-mm plate) were treated with YPFS for 48 h. Cell lysates were collected, diluted to equal protein concentration, and analyzed by western blotting. Representative western blots are shown. Values are normalized using the internal control GAPDH and expressed as % of control (untreated culture), in mean ± SEM, *n* = 3. * or ^#^, *p* < 0.05, ** or ^##^, *p* < 0.01, *** or ^###^, *p* < 0.001.

In order to evaluate YPFS on expression of ATPase, cultured A549-DDP cells were treated with YPFS for 48 h. Application of YPFS induced a distinct downregulation of protein expressions of P-gp and BCRP in dose-dependent manners, as compared with control group in cultured A549-DDP cells. An ∼25% decrease of P-gp and ∼80% decrease of BCRP were identified at maximal response (Figure 5). However, the effect of YPFS on protein expression of P-gp and BCRP in A549 cells was minimal (data not shown).

**FIGURE 6:**
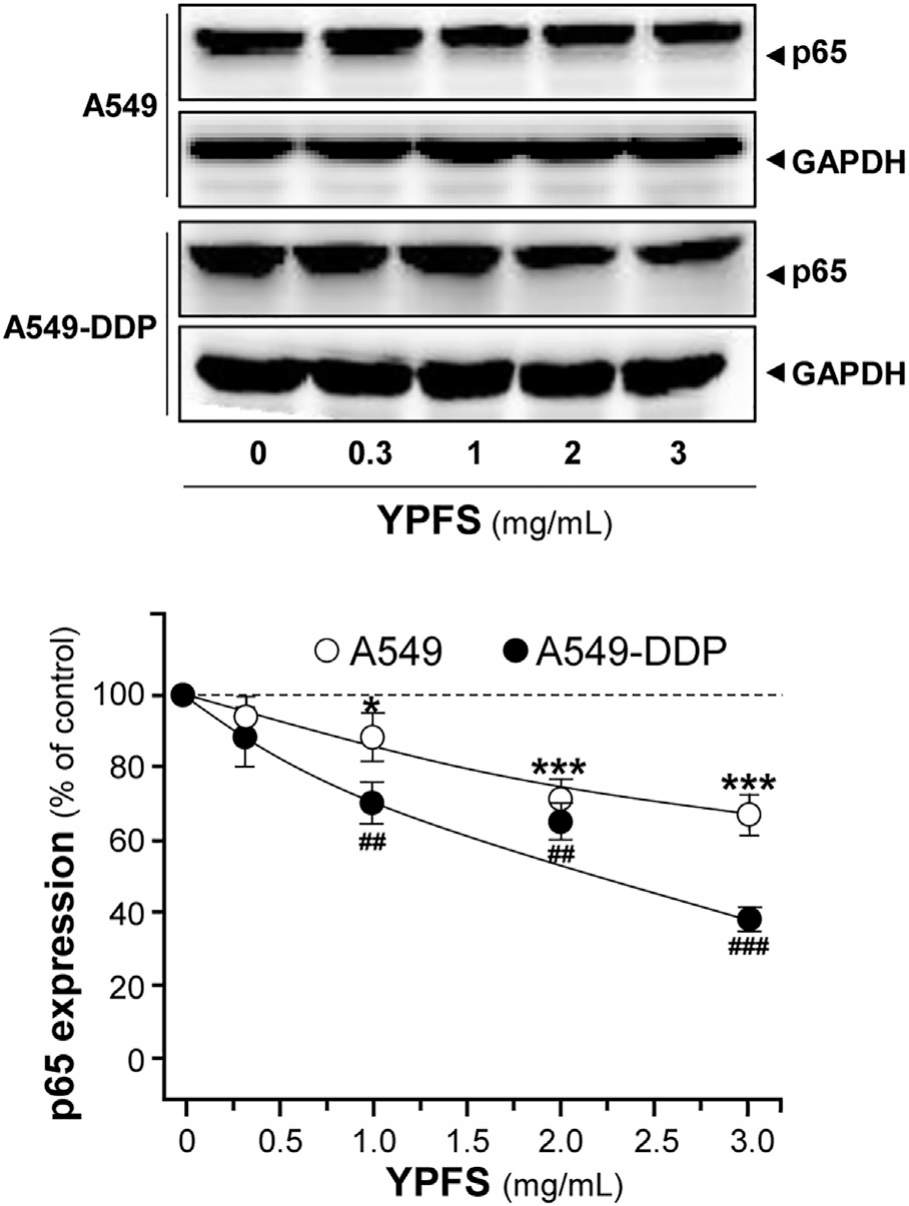
YPFS suppresses protein expression of p65 in cultured A549 and A549-DDP cells. Cultured A549/A549-DDP cells (120,000/well in 100-mm plate) were treated with YPFS for 48 h. Cell lysates were analyzed by means of western blotting as in Figure 4. Mean ± SEM are shown, *n* = 3. * or ^#^, *p* < 0.05, ** or ^##^, *p* < 0.01, *** or ^###^, *p* < 0.001.

NF-kB is known to activate a number of diverse genes in responding to stimuli, and one of the major members of NF-kB, p65, is known to be overexpressed in MDR cells. The role of p65 could exert pro-apoptotic or anti-apoptotic properties *via* regulating various targets, e.g. expressions of pro-apoptotic target Bax and anti-apoptotic target Bcl-2. The balance of Bax/Bcl-2 directly affects the rate of apoptosis. An obvious decrease of p65 expression in cultured A549 and A549-DDP cells was observed in a dose-dependent manner in presence of YPFS: the effects were reduced by ∼30% in A549 cells and ∼60% in A549-DDP cells (Figure 6).

**FIGURE 7:**
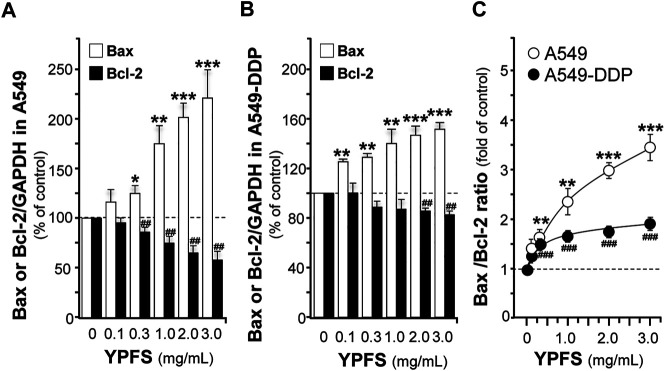
YPFS alters gene expressions of Bax and Bcl-2 in cultured A549 and A549-DDP cells. Cultured A549/A549-DDP cells (120,000/well in 6-well plate) were treated with YPFS for 24 h. The expression levels of target proteins were revealed by quantitative real-time PCR. The transcript levels of Bax and Bcl-2 in cultured A549 **(A)** and A549-DDP cells **(B)**. Bax/Bcl-2 ratio **(C)** in cultured A549 and A549-DDP cells. Values are in mean ± SEM, *n* = 3. * or ^#^, *p* < 0.05, ** or ^##^, *p* < 0.01, *** or ^###^, *p* < 0.001.

The apoptotic proteins were determined in YPFS-treated cultures. The mRNA of Bcl-2 was decreased; while Bax mRNA was increased in YPFS-treated cultured A549 and A549-DDP cells (Figures 7A,B). The expression levels of Bcl-2 were decreased by ∼40% (A549) and ∼20% (A549-DDP); while Bax levels were increased by ∼220% (A549) and ∼150% (A549-DDP) (Figures 7A,B). As a result, the ratio of Bax/Bcl-2 was increased to ∼3.3 folds in cultured A549 cells and ∼1.7 folds in cultured A549-DDP cells: both were in dose-dependent manners (Figure 7C). The results suggested an alternative way of YPFS in potentiating anti-cancer effect of cisplatin, targeting on p65 expression in cancer cells.

### Prim-O-glucosylcimifugin is One of the Active Ingredients in Yu Ping Feng San

**FIGURE 8:**
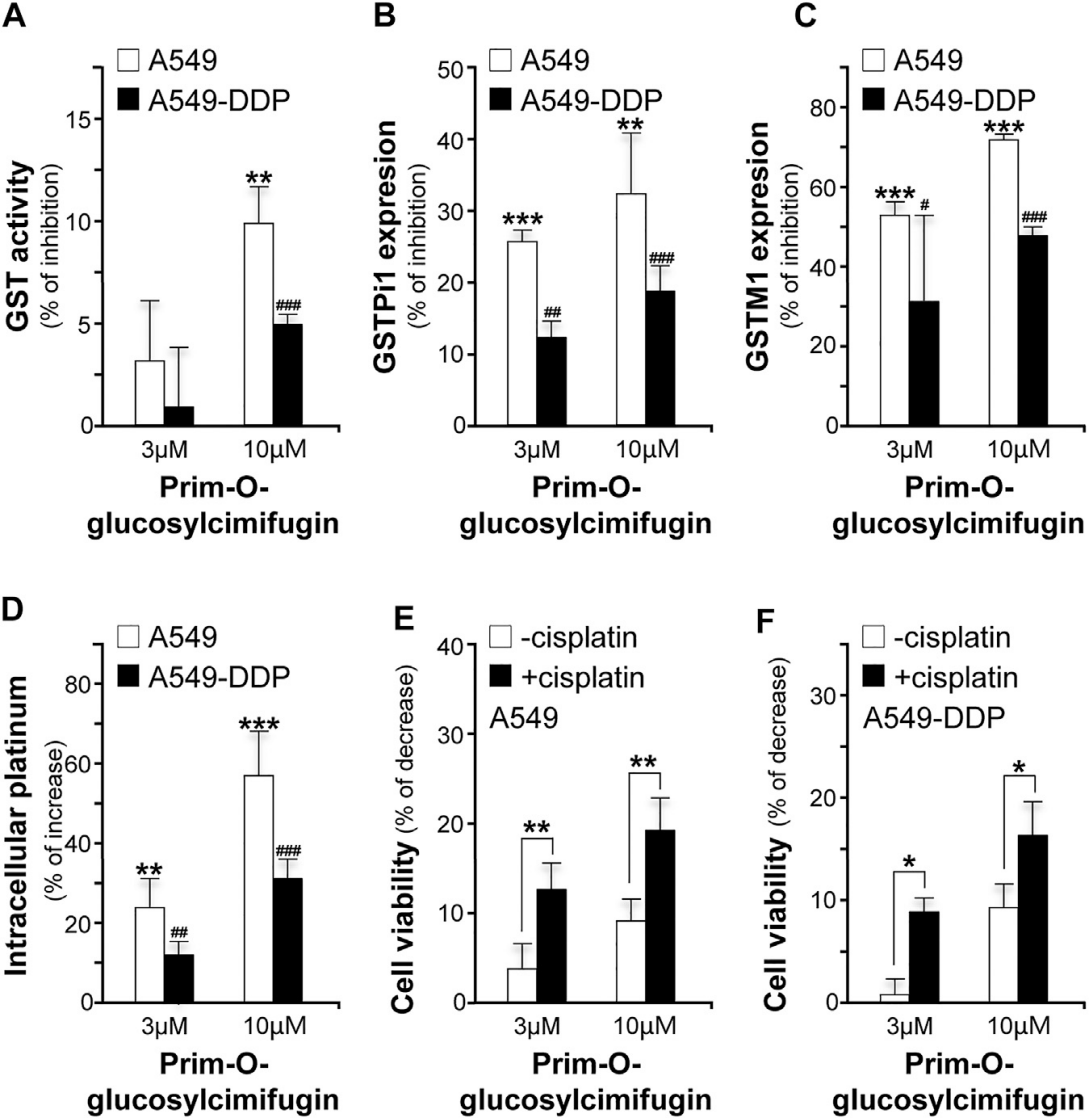
Identification of prim-O-glucosylcimifugin as an active ingredient of YPFS in reversing cisplatin chemo-resistance. Prim-O-glucosylcimifugin (3 and 10 µM) were applied onto cultured A549 and A549-DDP cells. Assays of GST activity (**A**), gene expression of GSTPi1 (**B**) and GSTM1 (**C**), measurement of intracellular platinum **(D)**, and cytotoxic activity (**E**, **F**) were performed and analyzed as described in Figures 1-3. Values are in mean ± SEM, *n* = 3. * or #, *p* < 0.05, ** or ##, *p* < 0.01, *** or ###, *p* < 0.001.

Prim-O-glucosylcimifugin, a major ingredient derived from SR and up to 0.882 mg/g in YPFS herbal extract, was subjected to evaluate its effects in reversing cisplatin chemo-resistance. The GST activities were decreased by ∼10% (A549) and ∼5% (A549-DDP) (Figure 8A); the expression levels of GSTPi1 were decreased by ∼33% (A549) and ∼18% (A549-DDP) (Figure 8B); the expression levels of GSTM1 were decreased by ∼70% (A549) and ∼50% (A549-DDP) (Figure 8C), and cisplatin accumulations were increased up to ∼56% (A549) and ∼30% (A549-DDP) (Figure 8D). Moreover, prim-O-glucosylcimifugin showed cytotoxic activity having ∼10% increase of cell death in A549 and A549-DDP cells (Figures 8E,F). The cytotoxic activities of prim-O-glucosylcimifugin, accompany with cisplatin, were increased up to two folds (A549) and ∼1.6 folds (A549-DDP) as that of prim-O-glucosylcimifugin (Figures 8E,F). The present results suggested that prim-O-glucosylcimifugin could be an active ingredient in YPFS in reversing the cisplatin drug resistance.

## Discussion

**FIGURE 9:**
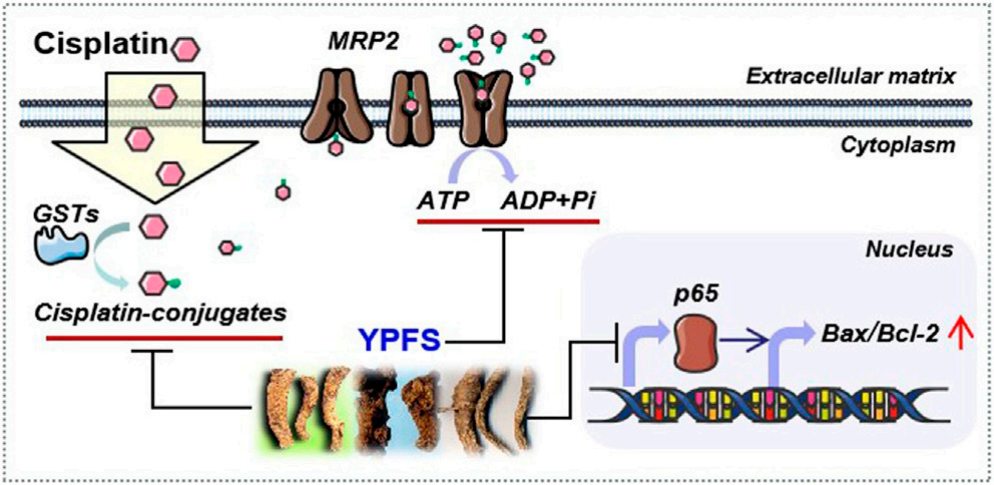
Model of YPFS in tracking the cisplatin‐resistance in a fast‐onset manner in lung cancer cells. YPFS reduces the activities and expressions of ATP‐binding cassette transporters and the levels of GSTs, consequently increases intracellular cisplatin in A549/A549‐DDP cells. In parallel, YPFS downregulates the expression of p65 subunit of NF‐κB leading to a higher ratio of Bax/Bcl‐2. Finally, YPFS synergize with cisplatin in triggering cell death.

Drug resistance remains a challenge in cancer therapy. Our previous study has found that YPFS could regulate p62/TRAF6 signaling (Lou et al., 2016), WT1/MVP axis and mTORC2/AKT signaling (Lou et al., 2018), which account the anti-MDR effects and anti-cancer effects both *in vivo and in vitro* models. To extend the function of YPFS in elevating the final intracellular cisplatin concentration in a fast-onset manner, we provided different lines of evidence of YPFS in reversing cisplatin-resistance in lung cancer cell lines by reducing the activities and expressions of ATP-binding cassette transporters, and the levels of GSTs. In parallel, YPFS downregulated the expression of p65 subunit of NF-κB leading to a higher ratio of Bax/Bcl-2. Besides, prim-O-glucosylcimifugin was identified as an active ingredient in YPFS to account in reversing cisplatin drug resistance. The final outcome of YPFS application is to synergize with cisplatin in triggering cell death, which therefore supports the notion of employing a Chinese herbal decoction in possible cancer treatment (Figure 9). In particular, this herbal decoction has been used for hundreds of years with well-known record of safety.

Lower bioavailability of cisplatin is another intractable problem, which limits the efficacy of cisplatin in chemotherapy. Many anti-cancer drugs are substrates for efflux transporters. Application of efflux transporter inhibitor is one of the strategies to reverse drug resistance; however, the usages of inhibitor for efflux transporter has been proven inefficient and severely toxic. None of these inhibitors have been developed for clinical application (Yang and Liu, 2016). The undeniable effect of Chinese medicine in reversing multidrug resistance has been demonstrated. For example, Sangeng mixture (Feng et al., 2003) and Shenghe powder (Wang et al., 2007) have been reported to downregulate the expression of P-gp transporter. In addition, Dahuang Zhechong pill was shown to reduce ATP level by suppressing the key metabolic enzymes, and the extract of *Rabdosia labtea* was shown to alter GST activity (Guo et al., 2020). As an outcome, these herbal extracts inhibited the activity of efflux transporters. In accordance to this notion, we provided several lines of evidence here to support the efficacy of Chinese medicine in against drug resistance during cancer therapy. The action mechanism of YPFS is to decrease final activity of efflux transporters, and which thereafter raises the bioavailability of drugs in cancer cells.

Consecutive activation of NF-κB is playing a role in immune response, including drug resistance of cancer cell (Li et al., 2013; Gong et al., 2017; Yu et al., 2018). Bcl-2 family, downstream targets of NF-κB including pro-apoptotic (e.g., Bax) and anti-apoptotic (e.g. Bcl-2) molecules, regulates the intrinsic pathway of apoptosis (Rózalski et al., 2005; Yu et al., 2018). Here, YPFS was shown to regulate p65 expression, as such to increase the ratio of Bax/Bcl-2, as well as the rate of apoptosis. In mitochondrial pathway, anti-apoptotic proteins of Bcl-2 family (e.g., Bcl-2) are acting on outer mitochondrial wall to prevent liberation of cytochrome c; while the pro-apoptotic proteins of Bcl-2 family (e.g., Bax) are migrating from cytosol to mitochondrial wall to increase the liberation of pro-apoptotic factors, particularly cytochrome c, resulting in cleavage of caspases, e.g., activating caspase-3 and caspase-9 (Xiong et al., 2016). In line to this notion, the role of YPFS in upregulating cleaved-caspase-3 and cleaved-caspase-9 have been illustrated (Lou et al., 2016). Here, we further provided evidence in supporting the regulation of YPFS in signaling of caspase-3 and caspase-9. Thus, the regulation of YPFS in intrinsic apoptotic pathway could serve as another underlying mechanism of YPFS in potentiating the chemosensitivity of cisplatin in cancer cells.

Prescribing of herbal formulae in treatment and prevention of diseases has been a key part of TCM practice, apart from the use of acupuncture and other manipulation treatments. The accumulation of experience gained over thousands of years has provided current application of TCM in situations, where conventional medicine has encountered difficulty, such as formation of drug resistance in chemotherapy of cancers, chronic diseases, and infections among other ailments. Chinese herbal medicine exhibits “multi-herb and multi-target” manifestation, which can be partially explained for its bi-directional actions and multi-targeting mechanisms. For example, pro- and anti-inflammatory effects of YPFS have been reported under different scenarios (Lee et al., 2007; Li et al., 2007; Du et al., 2013). The present investigations have provided another example of YPFS in having bi-directional effects. Apoptosis is initiated *via* intrinsic and extrinsic pathways. The enzyme dysregulating production of reactive oxygen species (ROS) changes cellular redox balance, and thus results in chemoresistance (Guo et al., 2020). ROS, a highly active molecule, has dual roles in accord to its level, i.e. normal level of ROS regulates the oxidative state for cell protection, but abnormal level of ROS increases the toxicity threshold resulting in DNA damage. The abnormal production of ROS leads to NF-kB translocation into nucleus and initiate pro-survival gene expression, which is one of the major causes for drug resistance (Parajuli et al., 2013). A previous report demonstrated that YPFS could potentiate cisplatin in enhancing ROS level in cultured lung cancer cells, leading to increase rate of apoptosis, whereas YPFS alone showed no effect in ROS production (Lou et al., 2016). Thus, the effects on ROS production in cisplatin-treated cultures could illustrate another bi-directional regulation of YPFS.

The synergistic effect of TCM could be identified in many cases, e.g., an 800-years old herbal decoction Dangui Buxue Tang having AR and Angelicae Sinensis Radix in a weight ratio of 5:1, produced better solubility and stability of the active ingredients, and the best biological activities in stimulating estrogenic responses, immunological responses, bone development and hematopoiesis function (Dong et al., 2006; Zheng et al., 2010). The present investigation supports the synergistic aspects of combined herbal ingredients in which the AR-derived calycosin and formononetin in YPFS could up-regulate activity of efflux transporter ATPase, and additionally compete the binding site of efflux transporters with cisplatin. Synergistic regime of calycosin, or formononetin, with cisplatin was found to elevate cisplatin accumulation in A549 cell resulting in higher apoptotic rate (Du, 2014). Thus, calycosin and formononetin within YPFS could be developed as competitive inhibitors for cisplatin in reversing the drug resistance. In addition to regulate GST, prim-O-glucosylcimifugin has been shown to exert anti-inflammation (Zhou et al., 2017), anti-allergic (Jia et al., 2019) and anti-cancer (Gao et al., 2019) effects. By having these lines of evidence, YPFS has provided us a strong motivation to develop the chemotherapeutic combined regime for anti-cancer treatment.

The integrative approach of YPFS could remarkably potentiate the effect of cisplatin within a short period of time *via* modulating the effects on GSTs and efflux transporter ATPases. Besides, the effects of YPFS in regulating intrinsic apoptotic signaling, including downregulation of p65 and upregulation of Bax/Bcl-2 ratio, were demonstrated here. This is an example of utilizing integrative medicine approach for difficulty cases, such as drug-resistance of cancer cell. Here, prim-O-glucosylcimifugin was identified as an active ingredient in YPFS to reverse cisplatin chemo-resistance. The aforementioned findings provided an understanding of the fast-onset action of YPFS in chemotherapy. The attractive identification of YPFS as a chemosensitizer in both *in vitro* and *in vivo* supports possible clinical application of this herbal medicine in cancer therapy. In line to this notion, AR showed anti-tumorigenic effects *in vivo*. The reduction of tumor volume, as well as pro-apoptotic and anti-proliferative effects, in HT-29 nude mice xenograft, treated with AR, was comparable with that produced by the conventional chemotherapeutic drug 5-fluorouracil (5-FU) (Tin et al., 2007). In the same investigation, the side effects (body weight drop and mortality) associated with the drug combo 5-FU and oxaliplatin were not induced by AR. These results indicate that AR could be an effective chemotherapeutic agent in colon cancer treatment, which may also be used as an adjuvant in combining with other orthodox chemotherapeutic drugs to reduce the side effects of cancer drugs. In addition, AMR (Ruqiao et al., 2020) and SR (Yang et al., 2020) were also found to possess anti-tumor activity. In conclusion, both YPFS and its single herbal ingredients offer potential for further development of new effective products for integrative medical treatment of cancers.

## Data Availability

The original contributions presented in the study are included in the article/Supplementary Material, further inquiries can be directed to the corresponding authors.

## Abbreviations

ABC efflux transporters, ATP‐binding cassette efflux transporters; A549, adeno-carcinomic human alveolar basal epithelial cells; AR, Astragali Radix; AMR, Atractylodis Macrocephalae Rhizoma; BCRP, breast cancer resistance protein; DDP, cisplatin; 5-FU, 5-fluorouracil; GSH, glutathione; GSTs, glutathione S-transferases; HPLC, High performance liquid chromatography; HRP, horseradish peroxidase; ICP-OES, inductively coupled plasma-optical emission spectroscopy; MDR, multidrug resistance; MRP2, multidrug resistance associated protein 2; MRPs, multidrug resistance associated proteins; NaOV, Sodium orthovanadate; NSCLC, non-small-cell lung cancer; PCR, Polymerase Chain Reaction; P-gp, P-glycoprotein; ROS, reactive oxygen species; RNA, Ribonucleic Acid; SR, Saposhnikoviae Radix; SDS-PAGE, sodium dodecyl sulfate-polyacrylamide gel electrophoresis; SCLC, small-cell lung cancer; TCM, traditional Chinese medicine; YPFS, Yu Ping Feng San.
