# Supplementary figures and images for: The Mechanisms of Yu Ping Feng San in Tracking the Cisplatin-Resistance by Regulating ATP-Binding Cassette Transporter and Glutathione S-Transferase in Lung Cancer Cells
Source: Front Pharmacol. 2021 May 28;12:678126. doi: 10.3389/fphar.2021.678126 (PMC8202081; doi:10.3389/fphar.2021.678126)

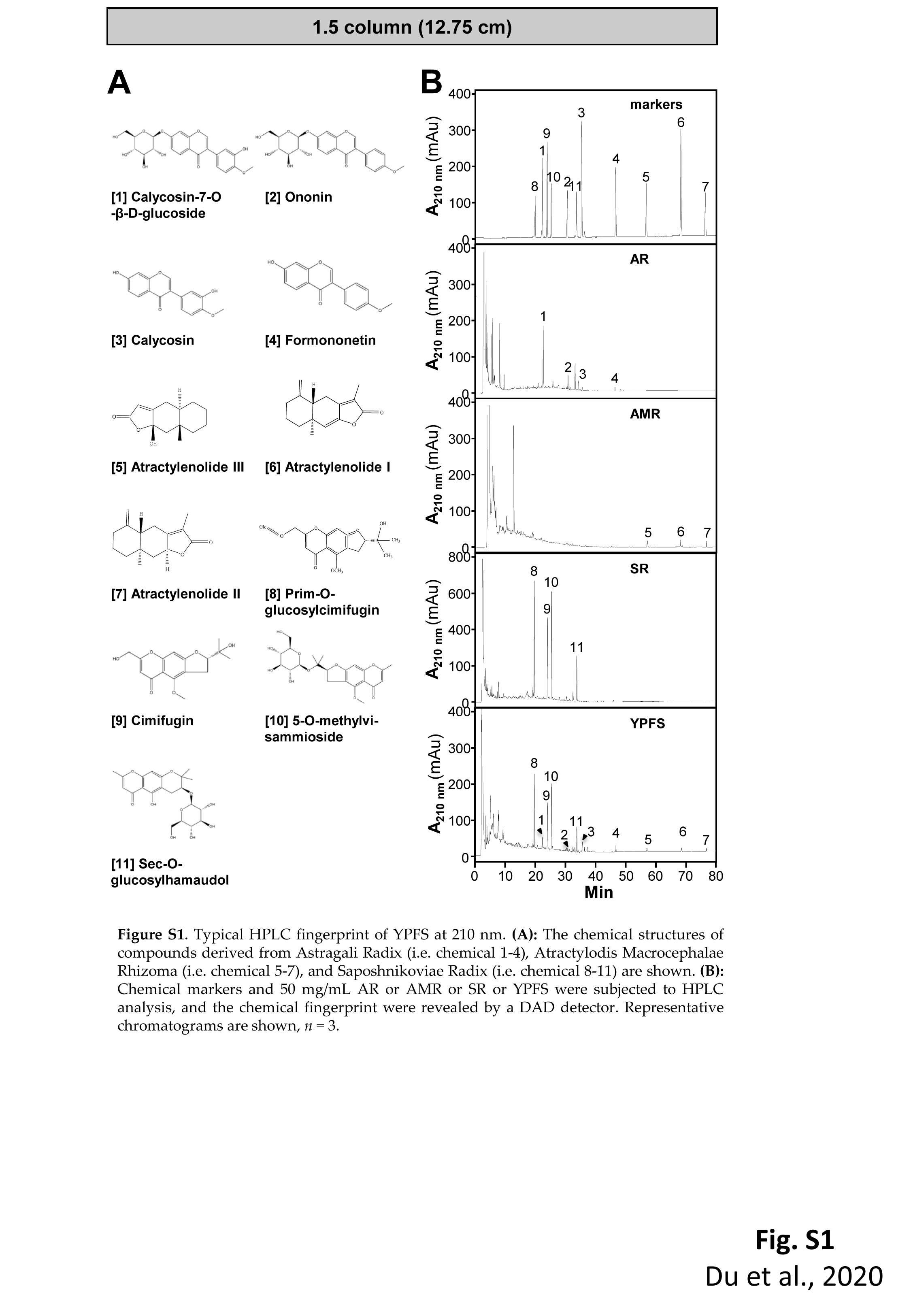

Supplement: Supplementary file 1 [file Image1.TIF]
